# Composition of mosquito fauna and insecticide resistance status of *Anopheles gambiae* sensu lato in Itang special district, Gambella, Southwestern Ethiopia

**DOI:** 10.1186/s12936-022-04150-5

**Published:** 2022-04-18

**Authors:** Tebiban Chanyalew, Gadisa Natea, Desalegn Amenu, Delenasaw Yewhalaw, Eba Alemayehu Simma

**Affiliations:** 1 Department of Biology, College of Natural Sciences, Jimma University, Jimma, Ethiopia; 2 School of Medical Laboratory Sciences, Faculty of Health Sciences, Institute of Health, Jimma University, Jimma, Ethiopia; 3 Tropical and Infectious Diseases Research Center, Jimma University, Jimma, Ethiopia

**Keywords:** Malaria, Insecticide resistance, *Anopheles* mosquito, Itang, Ethiopia

## Abstract

**Background:**

*Anopheles arabiensis*, member species of the *Anopheles gambiae* complex, is the primary vector of malaria and is widely distributed in Ethiopia. *Anopheles funestus, Anopheles pharoensis* and *Anopheles nili* are secondary vectors occurring with limited distribution in the country. Indoor residual spraying (IRS) and long-lasting insecticidal nets (LLINs) are pillars for the interventions against malaria control and elimination efforts in Ethiopia. However, the emergence and widespread of insecticide resistance in *An. gambiae sensu lato* (s.l.), might compromise the control efforts of the country. The aim of this study was to investigate composition of mosquito fauna and insecticide resistance status of *An. gambiae *s.l. in Itang special district ( woreda), Gambella, southwestern Ethiopia.

**Methods:**

Adult mosquitoes were sampled from September 2020 to February 2021 using the CDC light trap and pyrethrum spray catch (PSC). CDC light traps were placed in three selected houses for two consecutive days per month to collect mosquitoes indoor and outdoor from 6:00 P.M. to 06:00 A.M. and PSC was used to collect indoor resting mosquitoes from ten selected houses once in a month from October 2020 to February 2021. Moreover, mosquito larvae were also collected from different breeding sites and reared to adults to assess susceptibility status of populations of *An. gambiae *s.l. in the study area. Susceptibility tests were conducted on two to three days old non blood fed female *An. gambiae *s.l. using insecticide impregnated papers with deltamethrin (0.05%), alpha-cypermethrin (0.05%), propoxur (0.1%), pirimiphos-methyl (0.25%) and bendiocarb (0.1%) following World Health Organization (WHO) standard susceptibility test procedure. Molecular diagnostics were done for the identification of member species of *An. gambiae *s.l. and detection of knockdown resistance (*kdr*) allele using species specific polymerase chain reaction (PCR) and allele specific PCR.

**Results:**

In total, 468 adult mosquitoes were collected from different houses. *Culex* mosquitoes were the most dominant *(*80.4%*)* followed by *Anopheles* mosquitoes. Three species of *Anopheles* (*Anopheles coustani, An. pharoensis*, and *An. gambiae* s.l.) were identified, of which *An. coustani* was the dominant (8.1%) species. Higher number of mosquitoes (231) were collected outdoor by CDC light traps. Out of 468 adult mosquitoes, 294 were blood fed, 46 were half-gravid and gravid whereas the remaining 128 were unfed. WHO bioassay tests revealed that the populations of *An. gambiae *s.l. in the study area are resistant against alpha-cypermethrin and deltamethrin, but susceptible to bendiocarb, pirimiphos-methyl and propoxur. Of the total 86 *An. gambiae *s.l. specimens assayed, 79 (92%) successfully amplified and identified as *An. arabiensis*. West African *kdr* (L1014F) mutation was detected with high *kdr* allele frequency ranging from 67 to 88%.

**Conclusion:**

The detection of target site mutation, *kdr* L1014F allele, coupled with the phenotypic resistance against alpha-cypermethrin and deltamethrin call for continuous resistance monitoring.

## Background

In Africa, malaria is vector-borne disease caused by four *Plasmodium* species, namely *Plasmodium falciparum, Plasmodium ovale, Plasmodium malariae*, and *Plasmodium vivax.* The parasite is transmitted to human through the bite of infective female *Anopheles* mosquito [1]. There are over 445 recognized species of *Anopheles* mosquito of which around 70 species are potential malaria vectors [2]. The major vectors are *Anopheles gambiae* and *Anopheles funestus* species complexes, but there are also a number of primary and secondary vectors that contribute to the malaria transmission [2].

Approximately 60% of the Ethiopian populations live in malaria risk areas [3]. The disease primarily occurs up to the 2000 m elevation, but can also occasionally affect areas over 2000 m elevation in response to the spatial and temporal changes [4, 5]. Malaria transmission is unstable and seasonal which produces little immunity in the community; hence malaria epidemics are common and lead to high mortality and morbidity [3].

In Ethiopia, there are 47 documented Anopheles mosquito species [6], of which only four species are malaria vectors. *Anopheles arabiensis*, member species of the *An. gambiae* complex, is the primary vector of malaria widely distributed in the country [7, 8]. *Anopheles funestus, Anopheles pharoensis* and *Anopheles nili* are secondary vectors occurring with varying population densities, limited distribution and vector competence [3]. Very recently, an invasive *Anopheles* species, *Anopheles stephensi*, has been documented in the country [9], which might complicate the malaria elimination effort of the country [10].

Chemical based vector control intervention is the pillar strategy to combat malaria. Indoor residual spraying (IRS) and long-lasting insecticidal nets (LLINs) are instrumental for the significant reduction of malaria morbidity and mortality [11]. However, the emergence and widespread of insecticide resistance in the major malaria vectors might compromise effectiveness of chemical based (IRS and LLINs) interventions against malaria control and elimination efforts [12–17].

Insecticide resistance has become a serious challenge for the control and elimination of malaria due to the fact that malaria vectors are resistant to the four commonly used insecticide classes (pyrethroids, organochlorines, carbamates and organophosphates) [11, 17]. In Ethiopia, DDT resistance by *An. gambiae *s.l. was reported in the 1990s; since then widespread DDT resistance was documented throughout the country [12, 13, 18–21]. Moreover, resistance against other classes of insecticides, such as carbamates (bendiocarb), organophosphates (malathion) and pyrethroids (permethrin, deltamethrin) has been reported from various regions of the country [21, 22].

In the last decade, the number of malaria cases has declined due to a high coverage of IRS and scaling-up of LLINs [11, 17]. The national malaria control and elimination programme of Ethiopia has developed a malaria elimination road map to eliminate malaria from the country by 2030 [23]. However, this plan might be compromised as the magnitude of resistance against several insecticides is increasing in the *An. gambiae *s.l. populations [11, 21, 22].

Insecticide resistance largely caused by two major mechanisms. The first one is due to target-site insensitivity as a result of mutations in the target site of the insecticide that changes binding. The second mechanism is metabolic based resistance, where the insecticide is either degraded, sequestered or transported/excreted out of the cell before binding to the target site [24, 25].

Target-site and metabolic based resistance mechanisms operating in malaria vectors in several malaria endemic African countries have been documented. Knockdown resistance (*kdr*) is target site mutations in the voltage-gated sodium channel gene of mosquito nerve membranes is associated with DDT and pyrethroids resistance. In *Anopheles*, this involves the substitution of leucine (TTA) to phenylalanine (TTT) (*kdr* L1014F) or to serine (TCA) (*kdr*L1014S) [26, 27]. In addition, substitution of asparagine to tyrosine (N1575Y) is linked with resistance in *An. gambiae sensu stricto* (s.s.) [28], but not in *An. arabiensis* [21]. There is also an acetylcholinesterase gene (ace-1R) mutation, substitution of glycine (GGC) to a serine (AGC) which confers resistance to organophosphates and carbamates [29]. In Ethiopia, target site resistance mechanism, *kdr* L1014F (West Africa *kdr*), in populations of *An. arabiensis* documented in several populations across the country [12, 13, 19, 21, 22]. Metabolic based resistance in *Anopheles* mosquitoes has been reported from several countries in Africa [30–33]. Moreover, modifications in the cuticle either through cuticle thickening and/or altering of the cuticle composition of arthropods, which can slow down the penetration of chemical compounds [34–36] has also been reported in *Anopheles* populations [37].

Gambella is one of the malarious areas of Ethiopia with high malarial endemicity. Itang special district is known for its a stable form of malaria transmission [38]. Despite the current effort of the country, malaria incidence rate in Itang did not decline unlike many other malarious areas of Ethiopia [39]. Therefore, this study aimed to investigate the composition mosquito fauna and insecticide resistance status of *An. gambiae* s.l. in Itang, and detect target site mutations associated with DDT and pyrethroid resistance.

## Methods

### Study area

**Fig. 1:**
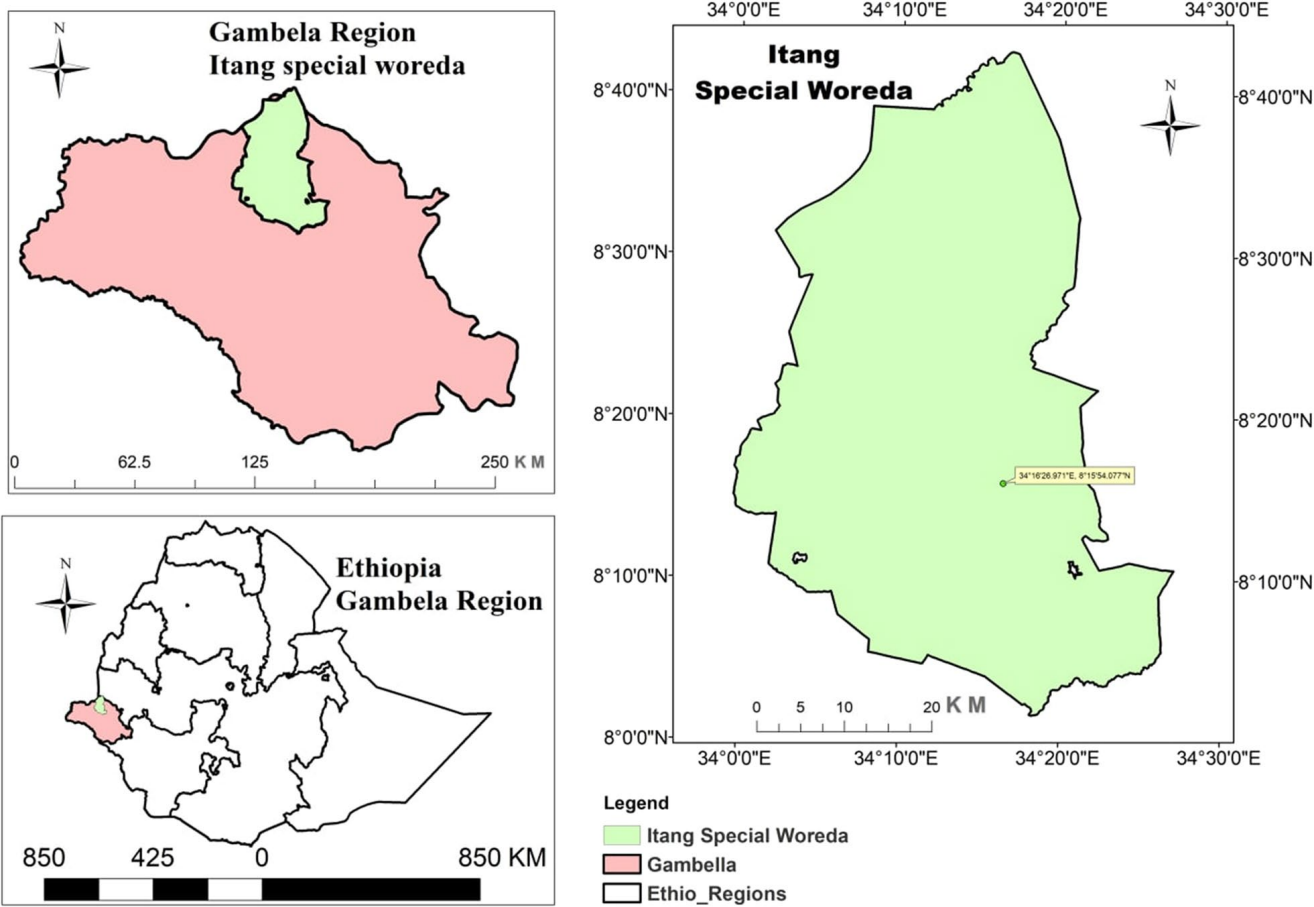
Map of the study area

Gambella is administrative regional state located in south western Ethiopia 777 km away from Addis Ababa (Fig. 1). Most of the region is flat, hot, and humid, with an altitude range of 300–2300 m above sea level and sloping westward. The annual average temperature of the region is 21.1–35.9 °C, with an average annual rainfall of 600 mm .The region has a total area of 25,802 km^2^ and administratively divided into three Zones (Nuwer, Agnua, Mezeng) and one special district, Itang [40]. According to the 2017 Ethiopian population projection, Gambella is sparsely populated with the total population approximately 436,000 [41].

The study was conducted from September 2020-Feburary 2021 in Itang special district, which is situated 42 km to the west of the regional capital of Gambella. Itang has 23 kebeles, with estimated total population of 45,772 and 9154 households [41]. The district’s average annual temperature and rainfall are 29 °C and 1000 mm, respectively. The climatic conditions of the district are favourable for the existence of a stable form of malaria throughout the year [38].

### Mosquito rearing

Potential mosquito breeding habitats (Baro river fringes, paddle or farming field ditches, sewerage, and stagnant water pools) were first inspected for the presence of mosquito larvae and positive habitats were sampled with a 350 ml capacity mosquito scoop. Dipping was done following World Health Organization (WHO) guidelines and standard operating procedures for entomological surveillances [42]. Anopheline mosquito larvae were sampled from various breeding sites, and kept in a room at constant 80% ± 10% RH and 27 ± 2 °C. The larvae were fed baking powder. The pupae were sorted and transferred to beakers and placed inside a cage adults to emerge. Adults were provided 10% sugar in the cage and, 2–3 days old female mosquitoes were used for insecticide susceptibility tests. The anopheline larvae collected from different breeding sites were not used for species composition analysis.

### Adult mosquito collection

#### CDC Light trap catches (LT)

Adult *Anopheles* were collected each month from September 2020 to February 2021 using CDC light traps from selected houses in study area. CDC light traps were placed in three selected houses indoor and outdoor from 6:00 P.M. to 06:00 A.M. to collect female *Anopheles* for two consecutive days per month from September 2020 to February2021. Due to scarcity of CDC light traps only three houses were selected for adult collections.

#### Pyrethrum spray catch (PSC)

In addition to CDC light traps, indoor resting mosquitoes were collected using PSC from ten selected houses from 6:00 A.M. to 9:00 A.M. once in a month from October 2020 to February 2021. Before spray the whole house was covered by white sheet and closed every opening such as windows, door, and others. After fifteen minute of spraying knockdown mosquitoes were collected and recorded and separately placed in Eppendorf tube. *Anopheles* were morphologically identified to the species using taxonomic keys [43, 44].

### Insecticide susceptibility test

Two to three days old non blood fed adult females morphologically identified as *An. gambiae *s.l. (from now on referred to as *An. gambiae*) were exposed to insecticide impregnated papers with discriminating doses of deltamethrin (0.05%), alpha-cypermethrin (0.05%) propoxur (0.1%) pirimiphos-methyl (0.25%), bendiocarb (0.1%) and control papers impregnated with oil. Insecticides were selected based on their current operational significance in the national malaria control programme. Pirimiphos-methyl, propoxur and bendiocarb are currently used for IRS in Ethiopia and pyrethroid is incorporated in LLINs. The insecticide impregnated and control papers were obtained from the WHO collaboration Centre, Vector Control Research Unit, School of Biological Sciences, Penang, Malaysia. The insecticides were first tested on susceptible strain *An. arabiensis* collected from Jimma University Tropical and Infectious Disease Research Center insectary to assure the quality of the impregnated papers. Then, the susceptibility test was carried out on non-blood fed female mosquitoes collected and reared from the study area. Batches of 25 mosquitoes in four replicates were exposed in test kit tubes with insecticide-impregnated papers and a control in two replicates, each with equal number of mosquitoes, exposed to papers impregnated with silicone oil was run in parallel for all bioassays for 1 h. Knockdown were recorded at 10, 15, 20, 30, 40, 50, and 60 min [45]. After 1 h, mosquitoes were transferred into holding tubes and provided with 10% sucrose solution with soaked cotton pads. Mortality was recorded 24 h post exposure. Mosquitoes, both dead and alive, were individually preserved in Eppendorf tubes over silica-gel for further molecular assays. The same numbers of mosquitoes were exposed to insecticide free papers as controls.

### DNA extraction of *An. gambiae*

Genomic DNA of individual *Anopheles* mosquito was extracted from 75 survived and 11 dead mosquitoes (sampled from mosquitoes exposed to alpha-cypermethrin and deltamethrin) using DNAzol reagent (MRCgene, USA) with minor modification of the protocol [46]. Due to limitations of reagents and consumables fewer samples of mosquitoes were used for the molecular assays.

### Molecular identification of *An. gambiae* s.l.

Morphologically identified *An. gambiae* female mosquitoes (alpha-cypermethrin and deltamethrin survived and dead mosquitoes) were selected for the molecular identification of members of *An. gambiae* species complex using species specific polymerase chain reaction (PCR) at Molecular Biology Laboratory, Tropical and Infectious Diseases Research Centre (TIDRC), Sekoru, Jimma University. *Anopheles arabiensis* susceptible colony strain was used as a positive control. The PCR assay was done adapting the established protocol [47]. In brief, PCR reaction was prepared for 20 µl final volume of 10 µl master mix, 0.5 µl of each primer *(*Universal primer *(*5′-GTGTGCCCCTTCCTCGATGT-3′), *An. arabiensis *(5′-AAGTGTCCT TCTCCATCCTA-3′), *An. amharicus *(5′-CAGACCAAGATGGTTAGTAT-3′)), 7.5 µl nuclease free water and 1 µl template DNA. The PCR program was set for an initial step at 94 °C for 10 min and then, for 30 cycles at 94 °C, 50 and 72 °C for 30 s each, respectively and a final extension step at 72 °C for 5 min. Finally, the band size of PCR products for each species was visualized on a 2% agarose gel.

### Detection of L1014F (*kdr* allele)

Allele specific PCR assay was conducted on the same specimens used for the identification of member species of *An. gambiae* s.l. The presence of West Africa *kdr* (L1014F) mutation was detected adapting the established protocols [26, 27]. In brief, the PCR was done with 25 µl final volume including 2.5 µl PCR buffer, 0.5 µl dNTP mixture, 0.75 µl MgCl, 0.8 µl Agd 1 (5′-ATAGATTCCCCGACCATG-3′) primer, 0.8 µl Agd 2(5′-AGACAAGGATGATGAACC-3′) primer, 1.5 µl Agd3*(*5′-AATTTGCATTACTTACGACA-3′) primer, 1.5 µl Agd4 (5′-CTGTAGTGATAGGAAATTTA-3′) primer, 0.15 µl Taq DNA polymerase and 1 µl DNA template to detect *kdr* L1014F allele. The following PCR program was set 94^°^C for 3 min, and 94 °C for 1 min, 52 °C for 30 s, 72 °C for 30 s for 40 cycles and a final extension step at 72 °C for 10 min. Finally, the band size of the PCR products for *kdr* allele was visualized on a 2% agarose gel to determine the genotype to homozygous resistant, heterozygous resistant and susceptible or wild type *kdr* allele. Due to reagents and scarcity of consumables more samples were not analysed. Moreover, East Africa *kdr* (L1014S) allele was not screened.

### Data analysis

Susceptibility status data was analysed based on the WHO 2016 classification criteria. As per the criteria 24 h mortality rate 98% and above considered fully susceptible; between 90 and 98%, possible resistance or suspected resistance; and mortality below 90% classified as resistant. When the control mortality was between 5 and 20%, the average observed mortality was corrected using Abbott’s formula [48]. When the control mortality was above 20%, the test result was discarded and the test was repeated.

## Results

### Mosquito densities and species composition

In total, 468 mosquitoes were collected using CDC light trap and PSC collection methods from different houses (Table 1). The majority of mosquitoes were *Culex* spp. (80.4%) followed by *Anopheles* mosquitoes. Three species of *Anopheles* mosquitoes, such as *Anopheles coustani, An. pharoensis*, and *An. gambiae *s.l. were identified, of which *An. coustani* was dominant among others (8.1%; n = 38) (Table 1). As shown in Table 1 the higher number of mosquito was collected outdoor by CDC light traps-night.

**Table 1:** Species composition and abundance of mosquitoes in Itang special district, southwestern Ethiopia from September 2020 to February 2021

Mosquito Species	CDC Light trap				PSC		Total	
	Indoor	(%)	Out door	(%)	No.	(%)	No.	(%)
*An. coustani*	10	5.4	26	11.2	2	3.9	38	8.1
*An. pharoensis*	8	4.3	24	10.4	3	5.9	35	7.5
*An. gambiae*	9	4.8	8	3.5	2	3.9	19	4
*Culex* spp	159	85.5	173	74.9	44	86.3	376	80.4
Total	186	100	231	100	51	100	468	100

### Abdominal status of different mosquito species

The abdominal conditions of all collected mosquito samples were classified into unfed, freshly fed, half gravid, and gravid. Out of 468 adult mosquitoes collected, 294 were fed while 46 were half-gravid and gravid (Table 2).

**Table 2:** Abdominal status of different mosquito species collected from the study area

Species	Blood fed	Unfed	Half gravid	Gravid	Total (%)
*An. coustani group*	9 (3%)	24 (18.8%)	4 (12.9%)	1 (6.7%)	38 (8.1%)
*An. pharoensis*	9 (3%)	19 (14.8%)	5 (16.1%)	2 (13.3%)	35 (7.5%)
*An. gambiae*	4 (1.4%)	11 (8.6%)	3 (9.7%)	1 (6.7%)	19 (4.1%)
*Culex* spp.	272 (92.6%)	74 (57.8%)	19 (61.3%)	11 (73.3%)	376 (80.3%)
Total	294 (100%)	128 (100%)	31 (100%)	15 (100%)	468 (100%)

### Insecticide resistance status of *An. gambiae *s.l. against different insecticides

As per WHO criterion, the local mosquito populations of *An. gambiae* s.l. were resistant to two groups of pyrethroid insecticides (deltamethrin and alpha-cypermethrin). Mortality rates of *An*. *gambiae* s.l. against deltamethrin and alpha-cypermethrin was 58% and 42%, respectively. However, the populations of *An. gambiae* s.l. were completely susceptible to pirimiphos-methyl, propoxur and bendiocarb, where 100% mortality rate was recorded for the three insecticides (Table 3).

**Table 3:** Insecticide susceptibility status of *An. gambiae *s.l. populations from Itang special district, southwestern Ethiopia, from September 2020 to February 2021

S.N	Insecticide	*An. gambiae* s.l tested			Phenotypic resistance
		No. tested	No. dead	% mortality	
1	Alpha-cypermethrin (0.05%)	100	42	42	Resistant
2	Deltamethrin (0.05%)	100	58	58	Resistant
3	Propoxur (0.1%)	100	100	100	Susceptible
4	Bendiocarb (0.1%)	100	100	100	Susceptible
5	Pirimiphos-methyl (0.25%)	100	100	100	Susceptible

### Molecular identification of *An. gambiae* s.l. and detection of L1014F *kdr* allele

Out of the total 86 *An. gambiae* s.l. samples assayed using species specific PCR, 79 (92%) of the specimens were successfully amplified and genotyped, and all were identified as *An. arabiensis.* The allele specific PCR revealed the presence of the knock-down resistance *(kdr*) L1014F allele with 92.7% (n = 51) homozygous and 7.3% (n = 4) heterozygous *kdr* resistance allele respectively with the *kdr* allele frequency ranging from 67 to 88% (Table 4).

**Table 4:** Genotypic and *kdr* allele frequency in populations of *An. arabiensis* from Itang special district, southwestern Ethiopia, from September 2020 to February 2021

Insecticide	Number assayed	Bioassay phenotype	Genotype			Allele frequency	
			SS	RS	RR	R	S
Deltamethrin	34	Survived	5	4	16	0.72	0. 28
	6	Dead	1	–	2	0.67	0.33
Alpha-cypermethrin	41	Survived	4	–	30	0.88	0.12
	5	Dead	1	–	3	0.75	0.25

RR: Homozygous resistant; RS: Heterozygous resistant; SS: homozygous susceptible; R: resistant; S: wild type

## Discussion

*Anopheles coustani, An. pharoensis* and *An. gambiae* s.l. were the three species identified from Itang, southwestern Ethiopia. Of all *Anopheles* mosquitoes, *An. coustani* was the most prominent species followed by *An. pharoensis* and *An. gambiae* s.l. In the study site, the abundance of mosquito species collected by CDC light trap was higher outdoor than indoor. Unlike many other localities in Ethiopia [49–51], the abundance of *An. coustani* dominated *An. gambiae *s.l. A study from Lare, Gambella documented higher density of *An. gambiae* than *An. coustani* [50], contrasting the result of the current study. This difference might be due to sampling period difference which leads to variation or shifts in density for various species of mosquitoes. Taye and his coauthors sampled mosquitoes from May to September 2017 whereas in our study sampling time was between September and February. Moreover, the difference might also be due to the type of breeding habitat. The breeding site where the *Anopheles* mosquitoes sampled was shore to the Baro River and due to this reason the breeding sites were covered by plants and shaded the water which might be favorable for *An. coustani* [52]. Very recently, higher density of *An. coustani* than *An. gambiae* s.l. populations were reported from non-irrigated swampy and river edges in Arjo Didessa, south west Ethiopia [53]. Unlike *An. coustani*, *An. gambiae* s.l. typically breeds in small, clear, sunlit temporary water pools [54] where vegetative cover is low [52, 55]. In Ethiopia the role of *An. coustani* in malaria transmission not clearly documented. Very recently, *An. coustani*, was found being infected with *Plasmodium* species [56] showing evidence of possible malaria transmission by *An. coustani* in the country. Therefore, the high abundance of *An. coustani* in Itang might call attention for further studies [57, 58].

The molecular identification of member species of *An. gambiae* complex confirmed that *An. arabiensis* is the only member species found in the study area. This finding is similar to previously reported studies from central, south west and southern parts of Ethiopia [10, 17, 19, 20]. *Anopheles arabiensis* populations from the study area were found highly resistant to deltamethrin like other populations of *An. arabiensis* from different localities of Ethiopia [10, 19, 20] Sudan [59] and Uganda [60]. The present study also showed that *An. arabiensis* populations were resistant to alpha-cypermethrin. Similarly, studies from South-West Ethiopia [61] and Congo [62] reported very high resistance to pyrethroids, but susceptible to bendiocarb, propoxur and pirimiphos-methyl. In contrast, populations of *An. arabinesis* form Malawi were found susceptible to alpha-cypermethrin [63].

The population of *An. arabiensis* collected from Itang found fully susceptible to bendiocarb and propoxur. This report is in agreement with other studies from different regions in Ethiopia [11, 19], Sudan [59], Yemen [64], Burkina Faso and Chad [65]. However, unlike the current finding, resistance against bendiocarb has been developed by *An. arabiensis* populations in some other parts of Ethiopia [17, 20]. The current result is similar to previously reported studies from different parts of Ethiopia [11, 66, 67] and Sudan [59] where propoxur fully killed *An. gambiae* s.l. However, in some other parts of Ethiopia resistance against propoxur has been observed [19, 20]. Similar to propoxur and bendiocarb the *An. arabiensis* populations from the study area were found susceptible to pirimiphos-methyl. This is similar with studies from different parts of Ethiopia [11, 19], Uganda [68] and Congo [62].

In this study, the population of *An. arabiensis* were screened for West African *kdr* allele (L1014F). A very high frequency of the West African *kdr *allele (L1014F), was observed with higher *kdr* allele indicating that target site resistance mechanism might contribute for the observed high level of pyrethroid (deltamethrin and alpha-cypermethrin) resistance in the population. This is similar with populations of *An. arabiensis* in some other areas of Ethiopia [11, 17, 19, 20]. The mutation, L1014F, is widespread in West and West Central Africa [69, 70] also becoming common in East African countries including Sudan [71]; Tanzania [72, 73] and Kenya [74, 75]. The L1014F *kdr* mutation in the voltage gated sodium channel is a target point mutation against pyrethroids and DDT [25, 76] which might occurred as a result of long and extensive use of DDT and pyrethroids for ITNs.

LLINs and IRS are the two most widely implemented malaria vector control interventions, and have resulted in a significant reduction of malaria-related mortality and morbidity in Ethiopia [9, 39, 77]. Universal access and coverage of LLINs by all household members regardless of age or gender [78] is not yet achieved in Ethiopia [78–80].

## Conclusion and recommendations

Overall, this study demonstrated that *An. coustani* was the predominant *Anopheles* species recorded among adult *Anopheles* mosquito collected during the study period in the study area. The high abundance of the species in the study area might call attention to further study the species for its role in malaria transmission. It also revealed that *An. arabiensis*, a member of *An. gambiae* complex, has developed high level of resistance against deltamethrin and alpha-cypermethrin. Moreover, target site mutation L1014F *kdr* allele with high frequency was detected in the populations.

For the success of malaria elimination from Ethiopia by 2030 [3], it is important to control the malaria vectors and focus on successful treatment. Therefore, understanding the type and density of malaria vector species in specific localities, their feeding behavior and interaction with humans is crucial for effective malaria vector control strategies. Moreover, evidence based insecticide susceptibility status of the malaria vector populations and understanding mechanisms of resistance operating in the populations is key for effective insecticide resistance management.

## Data Availability

Data used for this study are included in the manuscript.

## Abbreviations

CCEs: Carboxylcholinesterases
CDC: Center for Diseases Control and Prevention
CSA: Central Statistics Authority
DDT: Dichlorodiphenyltrichloroethane
DNA: Deoxyribonucleic acid
FMoH: Federal Minister of Health
GSTs: Glutathione S-transferases
ITNs: Insecticide-treated nets
*kdr*: Knock down resistance
LLINs: Long-lasting insecticidal nets
MSF: Medicins sans Frontières
OCs: Organochlorines
Ops: Organophosphates
P450s: Cytochrome-P450 monooxygenase
PSC: Pyrethrum Spray Catches
PY: Pyrethroids
SPSS: Statistical package for the social sciences
VGSC: Voltage gated sodium channel
WHO: World Health Organization
